# Management of “floating elbow” in children

**DOI:** 10.4103/0019-5413.33875

**Published:** 2007

**Authors:** SS Suresh

**Affiliations:** Ibri Regional Referral Hospital, PO Box 46, Ibri 516, Sultanate of Oman

**Keywords:** Floating elbow, forearm fracture, supracondylar fracture

## Abstract

**Background:**

Supracondylar fractures associated with ipsilateral forearm fractures, aptly termed as “floating elbow” is a rare injury in children after a fall from height. The various authors have reported their results with conservative treatment of one or both injuries to aggressive emergency operative fixation of both components.

**Materials and Methods:**

During a period of three years, the author managed four cases of floating elbow in children. All cases were managed by closed reduction and pinning of both components of the injury.

**Results:**

All patients recovered full elbow range of motion at three months followup and were rated as excellent as per modified Flynn's criteria. None of the patients developed cubitus varus deformity, complications related to the pins or delayed union.

**Conclusions:**

Early closed reduction and K wire fixation of both components of this injury gives better stability and prevents development of complications like compartment syndrome and elbow deformities.

## INTRODUCTION

Stanitski^1^ gave the term ‘floating elbow’ for a childhood injury when there is a fracture of the supracondylar region with ipsilateral fracture of the forearm bones. The treatment of this injury varies from conservative management of all injuries, single bone fixation to aggressive emergency fixation of all components. This being a very severe injury, usually following a fall from a height produces severe soft tissue damage which can result in neurovascular compromise or compartment syndrome. Immediate fixation of this fracture with percutaneous Kirshner wires to prevent complication and to avoid vascular complications has been reported to give good results. We report a series of four such cases.

## MATERIALS AND METHODS

All cases of supracondylar fractures of the elbow (Gartland Type III) requiring admission and closed pinning were reviewed over a period of three years (March 2004 to March 2007). There were four cases of fractures of the supracondylar area with ipsilateral fractures of one or both the forearm bones. Three patients were male and one female. The age of patients ranged from eight to 16 years with a mean of 11 years. In three cases the left upper extremity was involved. All cases were due to fall from a height more than two meters. All forearm fractures were distal at the metaphysis and displaced. All cases were extension type Gartland Type III supracondylar fractures. A case of supracondylar fracture with fracture of both the distal bones in a five-year-old girl was evacuated to a facility with a vascular surgeon due to suspected (and finally confirmed) brachial artery injury and hence is not included in the present report [Table 1].

**Table 1:** Showing the details of the patients

Age/Sex	Side	Mode of injury	Fracture pattern
16/M	Left	Fall from 2M	SC III/Distal radius
9/M	Left	Fall from tree	S/CIII/Distal both bones
11/M	Left	Fall from 2M	S/CIII/Distal both bones
8/F	Right	Fall from wall	S/CIII/distal radius

The closed reduction of supracondylar component was undertaken in the emergency room under intravenous sedation to reduce the soft tissue swelling and immobilized in a slab in extension. All patients underwent surgery within 24h of admission.

**Figure 1 (A-D):**
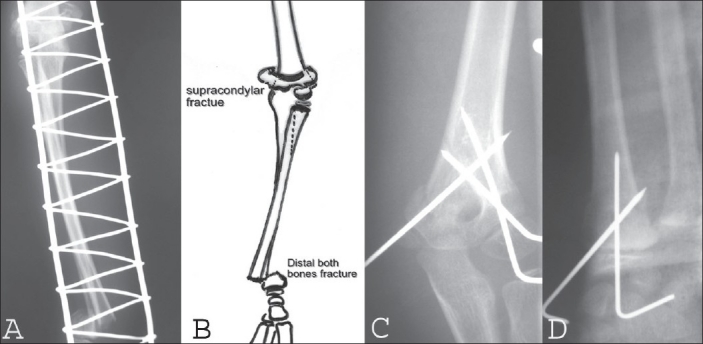
(A) X-ray of the elbow and forearm shows supracondylar fracture with ipsilateral fracture of both bone forearm in a 11 yrs old boy. (B) Line diagram of the same patient. (C) Post-operative X-ray of the elbow shows closed K-wire fixation of supracondylar fracture. (D) X-ray of the wrist shows closed percutaneous pinning of distal radius fracture

The forearm fractures were reduced, stabilized with percutaneous two or three crossed K wires, spanning the distal fragment to proximal. The supracondylar fractures were then reduced by longitudinal traction and manipulation and fixed with three Kirschner wires- two lateral-entry pins and a single medial entry pin. For lateral entry pins the pins are placed in the lateral and central columns of the distal humerus.^2^ The medial pin was passed with the elbow a little short of 90 degree flexion to prevent iatrogenic ulnar nerve injury. In one case with severe swelling of the elbow, the ulnar nerve was visualized through a mini incision and then the wire was passed. The pins were bent outside the skin to prevent migration and an above elbow slab was given. One case in which the above precaution was not taken developed iatrogenic ulnar nerve palsy which eventually recovered at three months followup [Figures 1 and 2].

**Figure 2(A-B):**
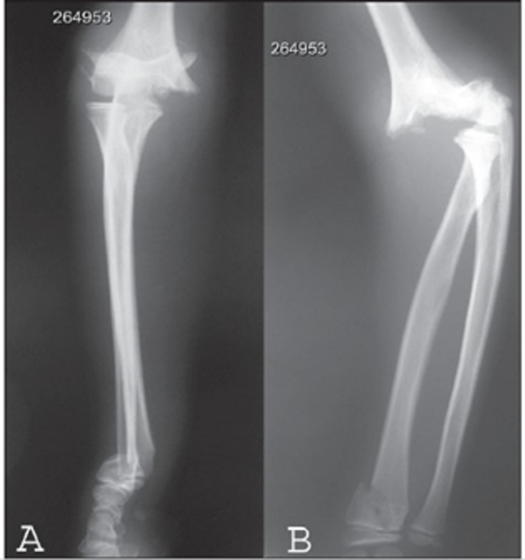
(A) X-ray of the elbow and forearm shows supracondylar with distal radius fracture. (B) Lateral view shows gross displacement of supracondylar fracture

**Figure 2 (C-E):**
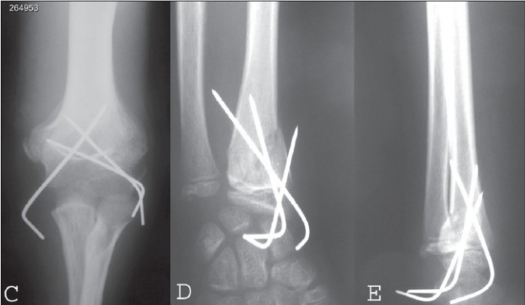
(C) AP X-ray of elbow shows precutaneous pinning of supracondylar fracture with callus formation. (D) AP and lateral X-ray (E) of distal forearm and wrist shows percutanous pinning of distal radius fracture

The supracondylar pins were left in place for six weeks and the forearm pins were removed at three weeks. Slab immobilization was continued for six weeks.

The mean duration of stay in the hospital was 4.75 days (four to six days).

The results were assessed by Flynn criteria as modified by Templeton and Graham [Table 2].^3^,^4^

**Table 2:** Modified Flynn criteria3,4

	Loss of elbow Flexion/extension	Loss of forearm pronation/supination	Loss of wrist flexion/extension	Change in carrying angle
Excellent	0°-5°	0°-15°	0°-15°	0°-5°
Good	6°-10°	16°-30°	16°-30°	6°-10°
Fair	11°-15°	31°-45°	31°-45°	11°-15°
Poor	>15°	>45°	>45°	>15°

## RESULTS

The patients were followed up at two weeks, six weeks and three months by the operating surgeon. The longest followup was two years and four months. None of the patients developed pin tract infection or myositis of the elbow. One girl in whom the medial pin was passed without necessary precaution developed iatrogenic ulnar nerve injury which recovered in three months time. None of the patients in this study developed compartment syndrome. None of the patients developed cubitus varus deformity. All patients recovered full elbow range of movements at three months followup and were rated as excellent as per modified Flynn's criteria.

## DISCUSSION

Reports of supracondylar fractures of the elbow with ipsilateral forearm fractures started appearing in English literature after the classic paper of Stanitski.^1^,^3^,^5^–^9^ Flynn *et al*,^4^ in their series of 331 supracondylar fractures reported one such case. It has been described as the most difficult combination to treat. Combination of supracondylar fractures with ipsilateral forearm fractures is rare with reported incidence of three to 17% of supracondylar fractures. A partially displaced extension supracondylar fracture with distal both bones forearm is the commonest association.^6^ Palmer *et al*,^2^ in their analysis of 78 supracondylar fractures found four ipsilateral fractures of the radius and ulna, two ipsilateral fractures of the radius alone and one ipsilateral midshaft ulna fracture. In the true sense floating elbow should include fracture supracondylar humerus with fracture of both bones forearm. However, various reports have included association of single bone fracture also in floating elbow.^1^,^3^,^5^

The chances of compartment syndrome are very high in such patients, due to the extensive soft tissue injury. The assessment of neurovascular status of the extremity is difficult due to extentive swelling. In a series by Blakemore^7^ the incidence of compartment syndrome was 33%.^9^

The maintenance of reduction is a problem with treatment with plaster alone. Due to severe edema the cast with elbow in acute flexion is hazardous because of the risk of ischemia. If the elbow is not flexed the chances of loss of reduction is high.^9^ In a retrospective review of 16 patients Ring *et al*,^7^ found two patients with compartment syndrome and four patients with incipient compartment syndrome in 10 patients treated with closed reduction and cast immobilization. None of the patients in their series who had percutaneous pinning of the fractures developed compartment syndrome.

There are many ways this fracture combination used to be managed ranging from conservative management to immediate surgical intervention. Reed in an early series (n = 15), treated all of them by conservative methods. Williamson and Cole^8^ treated these fractures with traction and delayed manipulation with percutaneous pinning if there was severe elbow swelling. Because of the difficulty in neurovascular monitoring the hospital stay is prolonged. Hence it is preferable to manage these injuries by operative intervention. Fowles (n = 175) reported six cases of this injury, all of them were managed by pinning of the supracondylar fracture and closed reduction and cast immobilization of the forearm fracture.^10^

Many methods are used for the management of the forearm fractures, ranging from closed reduction and casting, to percutaneous fixation to maintain reduction.^1^,^4^,^7^,^8^,^11^–^13^ The chances of displacement of the forearm fractures are high when immobilized in cast alone. Stabilization of the forearm fracture with precutaneous K wire ensures maintenance of reduction. Williamson and Cole^8^ managed the supracondylar fracture by traction or manipulative reduction and percutaneous pinning and the forearm fractures were managed by reduction and casting.

Roposch^4^ analyzed the results of pinning of the forearm fractures and compared this with closed reduction and casting. Three of his 18 patients with forearm fractures displaced in cast while none of the 29 cases pinned displaced. Biyani *et al*,^6^ maintained all forearm fractures in a cast and the supracondylar fracture was pinned. They stabilized the supracondylar fracture first with percutaneous wires if that fracture was found stable and then went on to reduce and immobilize the forearm fracture. Templeton and Graham treated the supracondylar fracture first, followed by reduction and stabilization of only radius.^3^

The forearm fractures were fixed first in a series by Tabak,^11^ followed by closed reduction and percutaneous fixation of the supracondylar fracture. This protocol was followed in the current series. Stanitski^1^ recommended closed reduction and transcutaneous wire fixation of the supracondylar fracture first followed by reduction and stabilization of the forearm fracture.

We used crossed pins, the medial pin passed without hyperflexion of the elbow and after palpating the ulnar nerve, to prevent iatrogenic ulnar nerve injury [Table 3]. In case of profound swelling a medial incision is made to locate the point of entry of the medial pin. Skaggs *et al*^14^ do not recommend routine use of medial pins to prevent iatrogenic ulnar nerve injury. Skaggs concluded that lateral pins alone give adequate fixation for unstable supracondylar fractures with the advantage of avoiding ulnar nerve injury.

**Table 3:** comparison of management and complication with different series.

Series	No of cases	Management of S/C fracture	Management of forearm fracture	Deformity	Compartment syndrome
Biyani 1989^6^	34	CR, rarely olecranon traction and pin	Closed reduction	7 cubitus varus	Nil
Templeton 1995^3^	8	CR pinning	CR pinning of radius	Nil	Nil
Harrington 2000^5^	12	CR pinning	All both bones pinned	Nil	Nil
Reed 1976^17^	15	CR and slab	CR and slab	Cubitus varus in 3/15	Nil
Roposch 2001^4^	47	Closed pinning	29- pinning	3/18 casted angulated	Nil
			18 -cast		
Ring 2001^7^	16	Closed pinning	10 cases- closed reduction only		6/10 compartment syndrome
			6 cases closed pinning		No cases in closed pinning
Tabak 2003^11^	23	Closed pinning	Closed pinning	Nil	Nil
Present study	4	Closed pinning	Closed pinning	Nil	Nil

Fowles^10^ used two pins laterally with good result, avoiding the iatrogenic ulnar nerve injury and better stability of the fracture. In a recent study by Skaggs *et al*,^14^ the authors found lateral pins alone effective in most unstable supracondylar fractures. It was necessary to stabilize the radius only in all cases, as the redisplacement after single bone stabilization is practically nil.^15^ Criteria of Flynn *et al*, modified by Templeton and Graham^3^,^16^ was used to assess the elbow function during followup. Harrington^5^ in a series of 12 children found 83% good or excellent results at the time of followup.

## CONCLUSIONS

Early anatomic reduction and fixation of both the supracondylar component and forearm fracture gives satisfactory cosmetic and functional results with a satisfactory outcome. Although the series is small to draw a conclusion it was presented to sensitize about this combination of injury.
